# Topological metrics as evolutionary and dynamical descriptors of conformational landscapes within protein families

**DOI:** 10.1371/journal.pcbi.1013985

**Published:** 2026-03-04

**Authors:** Nikhil Ramesh, S. Banu Ozkan, Eleni Panagiotou

**Affiliations:** 1 Department of Physics and Center for Biological Systems, Arizona State University, Tempe, Arizona, United States of America; 2 School of Mathematical and Statistical Sciences, Arizona State University, Tempe, Arizona, United States of America; Bogazici University, TÜRKIYE

## Abstract

Identifying the key order parameters that connect a protein‘s native structure to its dynamical and evolutionary behavior remains a central challenge. We introduce topological and geometrical metrics—specifically, writhe and Local Topological Energy (LTE)—to investigate these connections. Applying these tools to both present-day and ancestral forms of thioredoxin and *β*-lactamase, we show that LTE strongly correlates with established dynamical measures such as the Dynamical Flexibility Index (DFI). Remarkably, LTE distributions also track the evolutionary trajectories of these proteins, suggesting that the topological geometry of the native state encodes key aspects of both dynamics and evolution. Through molecular dynamics simulations, we further reveal critical shifts in the topological landscape of proteins, providing a molecular mechanism by which functional evolution proceeds via alterations in conformational dynamics. Extending our analysis to over 100 proteins, we provide the first compelling evidence that topological descriptors derived from static structures can reliably predict dynamical behavior. In general, our findings demonstrate that simple geometrical metrics capture essential features of protein conformational landscapes, offering a powerful new approach to bridging protein structure, dynamics, and evolution.

## Author summary

Advances in protein science and artificial intelligence have resulted in a wealth of protein structures, yet a fundamental challenge remains: how does a protein‘s native structure encode its dynamical behavior? Addressing this question is crucial for understanding protein function and advancing protein engineering, particularly for designing nanomachines. We introduce a novel approach, rooted in knot theory, to bridge this gap. We demonstrate that topological metrics, even when applied to static protein structures, reveal key aspects of protein dynamics. This discovery provides a rigorous and quantitative framework for predicting protein flexibility and evolutionary adaptation directly from structure. Our approach has broad implications, from improving AI-based protein modeling to anticipating functional outcomes of mutations, with applications in protein engineering, biotechnology and medicine.

## Introduction

The complex dynamics of proteins arise from a vast number of intra- and inter-molecular interactions and degrees of freedom, which are encoded, to an extent, by their structure. Molecular simulations show that structure-encoded dynamics indeed govern function [1–6]. Methods from mathematical topology offer a novel rigorous framework upon which protein structure complexity can be quantified in a way that is amenable to statistical and data analyses methods [7–16]. However, due to the mathematical nature of these methods, their physical relevance, beyond classification of native states, has been elusive. In this manuscript we show that the mathematical topology and geometry of the native state of proteins reflects aspects of protein evolution of function and dynamics.

Several mathematical approaches aim at characterizing and quantifying native 3-D structure of proteins by examining the relative positions of the constituent atoms [7–10,17,18]. Knot theory offers a different approach to understanding protein structure that accounts simultaneously for both relative positions of amino acids and their connectivity at all lengthscales [19–28]. With recent advances in topology and knot theory, we can now study all proteins beyond knotted proteins [12–16,20,22,29–32].

It has been challenging to establish a direct connection between the topological or geometrical complexity of a protein‘s static native structure and the broader topological and dynamical landscape of its conformational ensemble. This difficulty arises due to the lack of a well-defined order parameter or mathematical metric that can both compare static structures, and also accurately characterize the three-dimensional structural variability of those within the ensemble. Addressing this gap is particularly important as the traditional structure–function paradigm of protein activity is increasingly replaced by an ensemble-based model [1, 33–38]. In the ensemble model, the native state of a protein is not a single static structure but rather a collection of interconverting conformations that the protein explores [23,39]. The protein‘s function is governed by the dynamics of sampling these conformations, rather than being dictated by a single dominant structure [40,41]. This framework aligns well with protein evolution, as it explains how new functions can emerge or existing functions can be modulated for adaptation to new environments, all while maintaining the overall structural framework of the protein [42–50]. Computational methods provide a powerful means to assess functional changes and allosteric mechanisms that are inherently favored by the native ensemble [1–3,33–36,51]. One such approach, the dynamic flexibility index (DFI), offers a quantitative measure of these evolutionary mechanisms by linking structural dynamics to functional adaptation. This is achieved by applying linear response theory to quantify the response of each residue to external force perturbations. DFI has been successfully applied in various contexts, including establishing a broad relationship between the flexibility of individual amino acids and their evolutionary conservation [1,2,33–35,42,52–54]. This computational framework complements the ensemble model of protein dynamics by capturing how variations in flexibility profiles contribute to functional diversity while maintaining the overall native fold [55].

Building on the success of metrics like the dynamic flexibility index (DFI) in linking structural dynamics to functional evolution, we aim to develop topology-based metrics to assess functional evolution in proteins. One of the most intuitive mathematical measures of topological and geometrical complexity in proteins is the Gauss linking integral, which can be applied both to static X-ray crystallographic structures and to conformations sampled from molecular dynamics simulations. The Gauss linking integral is a measure of the number of times two curves wind around each other. When applied to one curve, it is called the writhe and it measures the amount of interwinding a curve does around itself. It is also often useful to compute the intercrossing number and average crossing number, which are coarser versions of these metrics. Previous studies have used the Gauss linking integral to quantify backbone entanglement by analyzing the entire protein backbone, examining the writhe of specific regions, or assessing linking between different structural elements [12–16,29,30,56,57]. These entanglement-based metrics, including the linking integral and other novel topological descriptors [58], offer significant advantages for studying protein conformational ensembles. As real-valued continuous functions of a protein‘s three-dimensional coordinates, they provide a mathematically rigorous framework for characterizing topological and geometrical features across different conformations. Prior research has demonstrated that nearly all proteins exhibit non-trivial topological and geometrical signatures, which are not only computationally identifiable but also physically meaningful, as they correlate with protein folding rates (at least for some datasets) [12,15,16,29,30,59,60]. By examining local conformations within structures deposited in the protein data bank, a measure known as local topological free energy was introduced [13]. This measure captures the rarity of specific local conformations in the folded state ensemble and has been shown to identify key mutation sites in proteins across diverse contexts [14,56,57]. Indeed, it has been shown that similar approaches integrated into machine learning has been successful for drug discovery [32].Thus, these topology-based metrics complement existing dynamical approaches like DFI, providing a new perspective on how structural complexity influences protein function and evolution. We stress that the LTE is not a physical energy, but a purely mathematical quantity that is normalized as an energy that depicts mathematically the conformational rarity of a local structure in the protein landscape. In this study, we employ mathematical topology and geometry to investigate how the evolution of the protein conformational ensemble reflects its dynamical evolution. Specifically, we apply the Gauss linking integral to both ancestral and extant native X-ray structures and their conformations sampled from molecular dynamics simulations, mapping these properties onto the evolutionary trees of thioredoxin and *β*-lactamase.

Thioredoxins are ubiquitous oxidoreductase enzymes found across all domains of life, from archaea to humans [61,62]. Over evolutionary time, they have adapted to environmental changes—such as variations in temperature and ocean acidity—by evolving toward reduced stability and decreased activity. In contrast, *β*–lactamases are bacterial enzymes that confer antibiotic resistance by hydrolyzing *β*–lactam antibiotics, including penicillin and cefotaxime (CTX), thereby inactivating them. The evolutionary trajectory of *β*–lactamases shows a transition from a generalist enzyme capable of degrading multiple antibiotics with moderate efficiency to a more specialized enzyme with enhanced activity against penicillins [34]. Our results indicate that, although the tertiary structure of ancestral and extant proteins remains largely conserved throughout evolution, the local topological free energy (LTE) effectively captures subtle local conformational changes. In the case of thioredoxins, LTE consistently decreases over evolutionary time, reflecting gradual structural adaptations. Notably, the LTE of static X-ray structures alone is sufficient to reconstruct the evolutionary tree of thioredoxins, highlighting its potential as a topology-based metric for evolutionary analysis.

By comparing LTE with dynamic flexibility index (DFI) profiles derived from conformational dynamics simulations, we observe a strong correlation between the LTE of static structures and their corresponding DFI profiles for both thioredoxins and *β*-lactamases. We also found this correlation to persist with a larger dataset of 100 proteins (S1 Data). This finding reinforces the connection between local geometric features of the native state and the broader dynamical landscape of proteins. Additionally, by leveraging topology and geometry, we examine the evolution of the topological landscape in molecular simulations. Our results reveal that, despite the overall conservation of tertiary structure, the topological landscape of ancestral and extant proteins differs, suggesting that topological features from knot theory (even in the absence of knotting) offer a distinct perspective on protein evolution and dynamics.

## Materials and methods

In this section we present the methods employed in this work to analyze the structure of proteins and their dynamics. More precisely, first, we present the definition of the Dynamic Flexibility Index, which examines the response of a protein under perturbation. Second, we present the metrics of topological structural complexity employed in this work, which are based on the Gauss linking integral.

### DFI

In this work, to study the dynamics of proteins we used a metric known as the dynamic flexibility index (DFI) [1,52]. The DFI captures the dynamical behavior of proteins by calculating the responses of each residue to perturbations. To calculate these responses DFI utilizes linear response theory (LRT) [2,63,64]. The underlying model for the protein used in DFI calculations is either taken to be an elastic network model (ENM) [65] or is obtained from covariance matrices from all-atom molecular dynamics simulations. The response is calculated from LRT using Eq 1. To ensure an isotropic response this response is averaged over multiple directions.


$$\Delta [R{]}_{3N\times 1}=[H{{]}^{-1}}_{3N\times 3N}[F{]}_{3N\times 1}$$ (1)

Here *N* refers to the number of residues in the protein, *H* is the Hessian matrix and it can be computed directly from the atomic coordinates. If the DFI is calculated using ENM, then this Hessian matrix is obtained from the second derivatives of the harmonic potential with respect to the cartesian coordinates. The hessian can also be extracted directly from all-atom molecular dynamics simulation as the inverse of the covariance matrix.

Using Eq 1 we can perturb each residue sequentially to obtain a perturbation response matrix *A*, given by,


$$\begin{array}{c} A=[\begin{array}{ccc} |\Delta R^{1}{|}_{1} & \ldots & |\Delta R^{N}{|}_{1}\\ \vdots & \ddots & \vdots \\ |\Delta R^{1}{|}_{N} & \ldots & |\Delta R^{N}{|}_{N} \end{array}] \end{array}$$ (2)

where $|\Delta R^{j}{|}_{i}$ is the response at residue number *i* due to a perturbation at residue *j*. We can then define the DFI at position *i* to be,


$$DFI_{i}=\frac{\sum_{j=1}^{N}|\Delta R^{j}{|}_{i}}{\sum_{i=1}^{N}\sum_{j=1}^{N}|\Delta R^{j}{|}_{i}}$$ (3)

The DFI quantifies the relative response to perturbations observed at any position. Residues with low DFI do not exhibit large fluctuations to perturbations and hence are more dynamically stable. These positions also tend to be evolutionarily conserved as they typically play a crucial role in the dynamics of these molecules [1].

### Topological parameters

A measure of conformational complexity of two curves in 3-space is the Gauss linking integral [66], which is defined as follows: for two disjoint oriented curves $l_{1}$ and $l_{2}$ with arc-length parametrizations $\gamma_{1}$ and $\gamma_{2}$ respectively, the *linking integral*, *Lk*, is the double integral over $l_{1}$ and $l_{2}$:


$$Lk(l_{1},l_{2})=\frac{1}{4\pi }\int_{\left[0,1\right]}\int_{\left[0,1\right]}\frac{\left(\dot{\gamma_{1}}(t)\times \dot{\gamma_{2}}(s))\cdot (\gamma_{1}(t)-\gamma_{2}(s)\right)}{||\gamma_{1}(t)-\gamma_{2}(s)|{|}^{3}}\;dtds$$ (4)

where $\dot{\gamma }$ denotes the derivative of *γ* and where the integral runs over [0,1]×[0,1], which denotes all s,t∈[0,1].

The Gauss linking integral is a measure of the number of times two curves wind around each other and can have both positive and negative values depending on orientations of the curves. When the two curves are closed (thus form a topological link), the Gauss linking integral is an integer-valued topological invariant. When the curves are open, it is a real-valued continuous function of the curve coordinates. The linking integral may be non-zero even for curves that do not visibly interwind. In those cases, it captures aspects of their relative positions related to their orientations and vicinity, which can be interpreted as topological interactions or a potential for interwinding. The Gauss linking integral can be expressed in terms of properties of link diagrams. An oriented link diagram is a projection of a pair of oriented curves to a plane, where double points keep the information of over/under and each crossing is labeled as a positive crossing (+1) or a negative crossing (−1) based on the relative orientations (see S1 Fig). A positive crossing and a negative crossing are also referred to as a right-handed crossing and a left-handed crossing, respectively. The linking integral is then the average of half the algebraic sum of signs of all crossings in a projection of two curves over all possible projection directions. For polygonal curves, the linking integral can be expressed as a finite sum of signed geometric probabilities that two edges cross in any projection direction.

When taking the absolute value in the integrand, we obtain the *intercrossing number (ICN)* between two curves, which measures the average number of crossings seen between two curves in any projection direction. Namely, for two disjoint oriented curves $l_{1}$ and $l_{2}$ with arc-length parametrizations $\gamma_{1}$ and $\gamma_{2}$ respectively, the *intercrossing number*, *ICN*, is the double integral over $l_{1}$ and $l_{2}$:


$$ICN(l_{1},l_{2})=\frac{1}{4\pi }\int_{\left[0,1\right]}\int_{\left[0,1\right]}\frac{\left|(\dot{\gamma_{1}}(t)\times \dot{\gamma_{2}}(s))\cdot (\gamma_{1}(t)-\gamma_{2}(s))\right|}{||\gamma_{1}(t)-\gamma_{2}(s)|{|}^{3}}dtds$$ (5)

where $\dot{\gamma }$ denotes the derivative of *γ* and where the integral runs over [0,1]×[0,1], which denotes all s,t∈[0,1].

The ICN is not a topological invariant, even for topological links and is more sensitive on the geometry of the conformations. It can be an advantageous metric when interested in capturing subtle geometric entanglement in complex systems.

The Gauss linking integral can also measure the degree at which a curve interwinds around itself. When applied to one curve, the Gauss linking integral is called the *writhe* of the curve; for an oriented curve *l* with an arc-length parametrization *γ*, the *writhe*, *Wr*, is the double integral over *l*:


$$Wr(l)=\frac{1}{2\pi }\int_{[0,1{]}^{*}}\int_{[0,1{]}^{*}}\frac{\left(\dot{\gamma }(t)\times \dot{\gamma }(s))\cdot (\gamma (t)-\gamma (s)\right)}{||\gamma (t)-\gamma (s)|{|}^{3}}\;dtds$$ (6)

where $\dot{\gamma }$ denotes the derivative of *γ* and where the integral runs over $[0,1{]}^{*}\times [0,1{]}^{*}$, which denotes all s,t∈[0,1] such that s≠t.

The writhe is not a topological invariant, even when the curve is closed (thus forms a knot). It is real valued continuous function of the curve coordinates that measures the number of times a curve winds around itself. Even though a high absolute writhe value may indicate topological complexity, the writhe is sensitive to local geometrical entanglement. The writhe can also be expressed as the average algebraic sum of signs of all crossings in a projection of a curve with itself over all possible projection directions.

The writhe, linking number, intercrossing number can be computed exactly, avoiding numerical integration, using the algorithm described in [67,68].

**Fig 1:**
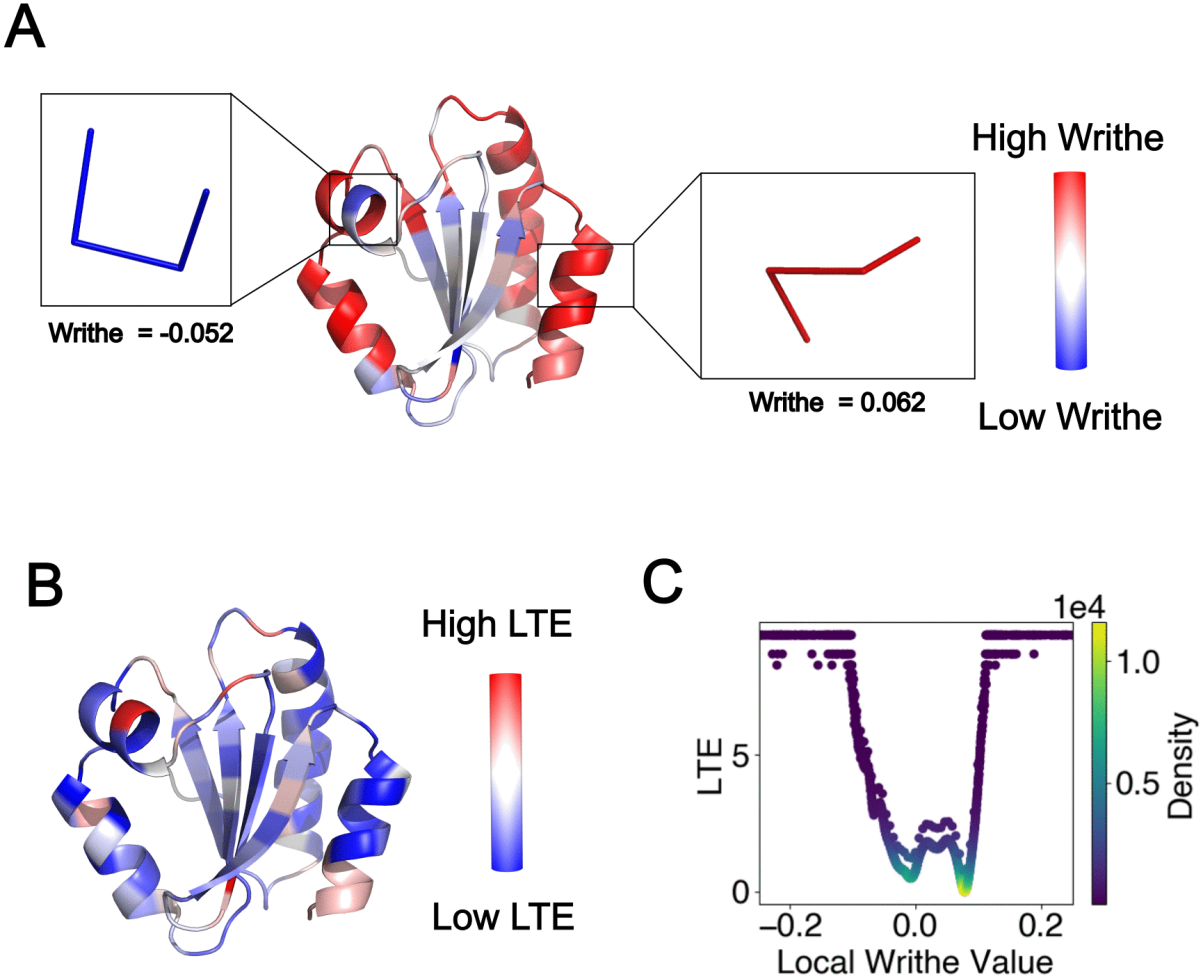
Local writhe and LTE of a protein. (*A*) shows the local writhe computed from the positions of four consecutive C*α* atoms along the chain of the human thioredixin (PDB ID: 1ERU). The local writhe values can be positive and negative, highlighted in blue and red, respectively. Examples of local conformations of the C*α* backbone corresponding to high positive and negative writhe highlighted. The local Writhe can take values between -0.5 and 0.5. High in absolute value writhe indicates tighter local conformations, while a value of zero indicates a straight conformation. The LTE along the chain for the same protein is shown in **(*B*)**. The variation of LTE with writhe as computed from a culled PDB sample dataset is shown in **(*C*)**. The LTE captures the rarity of local writhe value in the the PDB, with high LTE values indicative of rare local conformations.

An important property of the Gauss linking integral is that it can be applied to polygonal curves of any length to characterize 3-dimensional conformations at different length scales. In this manuscript we represent proteins by their C*α* atoms and compute the writhe of entire proteins, as well as that of parts of the protein at the length scale of 4 residues, which we call the *local writhe*. We assume an orientation of the resulting polygonal curves implied by their sequence. See Fig 1A for an example of local writhe distribution along a protein. We also apply the ICN to examine the relative conformations of parts of a protein. In particular, we apply this to proteins to examine the relative conformations of two helices with respect to the rest of the protein.

#### The local topological free energy in writhe.

The local topological free energy, originally defined in [13], can be used to assign a measure of rarity of local conformations along a protein backbone, relative to local conformations in the PDB. To do this in practice, we use a curated subset of the crystal structures provided in the PDB. Namely, we use the dataset of unbiased, high-quality 3-dimensional structures with less than *60*% homology identity from [69]. Then for each residue of a given protein we compare its local writhe value to those of the ensemble and a free energy is assigned to the residue based on the population of that value in the ensemble. More precisely, let $m_{Wr}$ denote the maximum occurrence value for the local writhe in the folded ensemble. To any value *p* of local writhe, we associate a purely topological/geometrical free energy, the local topological free energy (LTE):


Π(p)=ln[d(m)/d(p) (7)

where d(m),d(p) are the number of occurences of each value.

Fig 1B shows the LTE distribution as a function of local writhe values in the PDB sample used in this study.

The LTE has been used to successfully predict sites of interest in proteins in different contexts, such as mutation sites that may impact the conformational landscape of proteins and binding sites [13,14,56,57].

A summary of the topological/geometrical metrics and abbreviations used in this manuscript is given in Table 1 and further examples are given in the SI.

**Table 1:** The topological/geometrical metrics used in this manuscript with their abbreviation and specific usage herein.

Topological Metric	Abbreviation	Usage in this manuscript
local writhe	*Wr*	geometrical complexity of single
		of single conformations of
		4 consecutive C*α* atoms
local topological energy	LTE	a measure of rarity of a local
		writhe value as statistically determined
		using a non-redundant protein dataset
intercrossing number	ICN	pairwise
		geometric complexity of the conformation
		of a secondary structure element
		in a protein with the rest of the protein

## Results

### The evolution of the local topological landscape of proteins

Numerous studies on ancestrally resurrected proteins have demonstrated that, while protein function adapts and evolves over approximately three billion years of evolution, the three-dimensional structure remains largely conserved [1,42]. Indeed, the root-mean-square deviation (RMSD) between ancestral proteins and their corresponding extant homologs is typically less than 1 Å, indicating that these structural metrics alone do not provide meaningful insights into functional evolution. Therefore, applying our topological metrics based on knot theory to ancestral protein structures and their modern counterparts presents a promising framework for assessing functional divergence. In this study, we analyzed the functional evolution of two distinct enzyme families: *β*-lactamases, which evolved from substrate generalists to substrate-specific enzymes, and thioredoxins, which adapted to low-temperature and high-pH environments.

**Fig 2:**
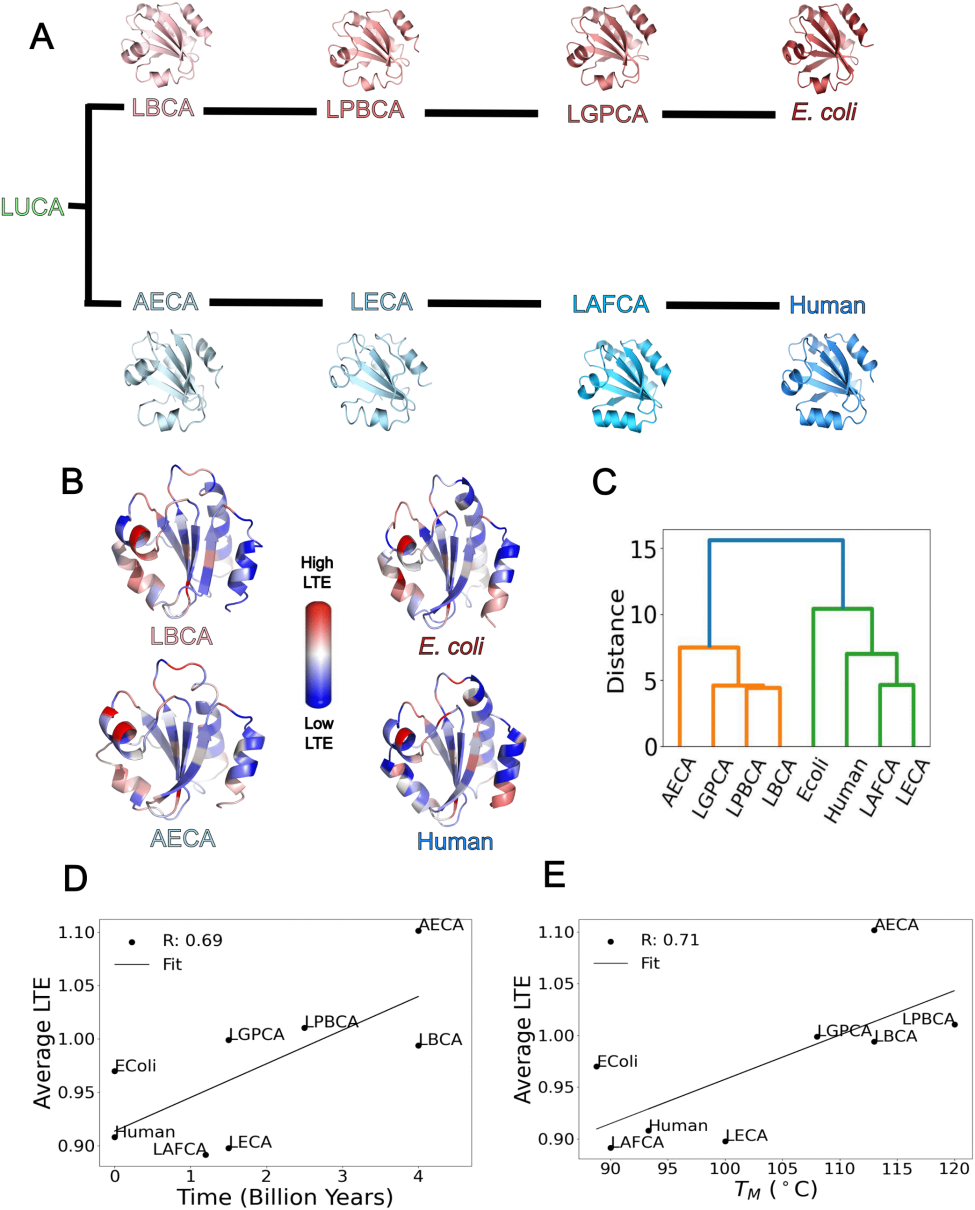
Topological evolution of thioredoxin. (*A*) shows the *E. coli* and Human branches of the thioredoxin phylogenetic tree (The PDB Codes are: LBCA - 4BA7, AECA - 3ZIV, LACA - 2YNX, LPBCA - 2YJ7, LGPCA - 2YN1, LECA - 2YOI, LAFCA - 2YPM, ***E.*** *Coli* - 2TRX, Human - 1ERU). In (*B*) we see the LTE profile colored on the crystal structures of the extant and the most ancient along both the branches. We see in (*C*) that a principal component analysis (PCA) of the LTE profiles shows that the two branches mostly cluster together. In (*D*) and (*E*) we see that LTE has a high correlation with both the evolutionary time of emergence of each variant and the melting temperature $T_{M}$. The reported *R* value corresponds to the Pearson correlation coefficient. The *p*-values for the correlations between the average LTE and (i) the evolutionary time of emergence and (ii) the melting temperature are 0.0566 and 0.0470, respectively. Evolutionary emergence times for each variant were taken from Ingles *et al.* [43], and melting temperatures were obtained from Risso *et al.* [70].

Fig 2A presents the evolutionary tree of thioredoxin, illustrating the divergence of ancestral lineages leading to modern homologs in *E. coli* and humans. The reconstructed ancestral proteins (LBCA, LPBCA, LGPCA, AECA, LECA, LAFCA) and modern homologs (*E. coli* and human) retain a conserved fold despite evolutionary divergence. The LTE profiles, depicted in Fig 2B highlight variations in LTE values across ancestral and extant structures, in particular the regions of *α*-3 and *α*-4 helices contributing stability and folding of the thioredoxins. Similarly, we also observed a difference in LTE values between ancestral *β*-lactamase (GNCA) and its modern homolog (panel A of S2 Fig). When we cluster the LTE profiles of both ancestral and extant thioredoxins in both lineages using principal component analysis, ancestral and extant homologs within each evolutionary branch tend to cluster together (Fig 2C). The clustering of the LTE profiles was performed using Ward‘s linkage method. We retained the top seven eigenvalues for this analysis because they account for over 90% of the variance in the data. Clustering of LTE values of ancestral *β*-lactamases with extant homolog yielded similar results, clustering ancestor gram positive and gram negative common ancestor (PNCA) and gram negative common ancestor (GNCA) *β*-lactamases together (panel B of S2 Fig). This clustering suggests a conservation of local energetic landscapes along evolutionary trajectories. To further investigate, we plot the average LTE of each homolog against its estimated time of emergence (Fig 2D). A positive correlation (R = 0.69) reveals that despite the high structural similarity between ancestral and extant thioredoxins, topology/geometry-based structural metric, (LTE) correlates with evolutionary time, effectively capturing functional evolution. This pattern is further reinforced in Fig 2E, which shows a strong correlation (R = 0.71) between LTE and the melting temperature ($T_{m}$). Overall, these results suggest that protein evolution favors local structural conformations that are, on average, more prevalent in the native protein structure ensemble, potentially enhancing functional robustness.

Analysis of how flexibility profiles evolved during the functional divergence of these enzymes revealed that modern homologs exhibit increased flexibility in specific regions compared to their ancestral counterparts [1,34,35]. This variation in flexibility is closely linked to protein stability and function [35]. Specifically, DFI analysis revealed that evolutionary substitutions tend to increase the flexibility of previously rigid hinge sites, while compensatory changes introduce new hinge points by reducing the flexibility of other regions. This “hinge-shift” mechanism fine-tunes protein dynamics, allowing adaptation to new functions and environments [1,71].

**Fig 3:**
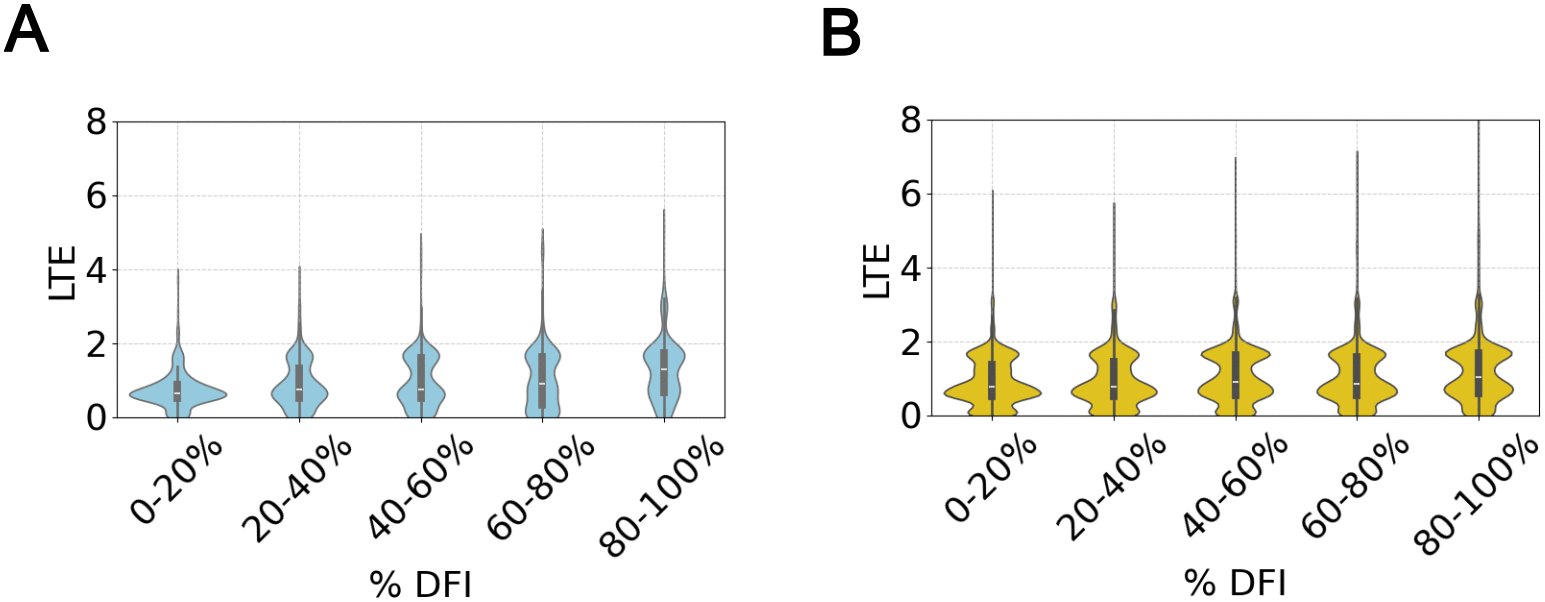
LTE is positively correlated with DFI. On binning the percentile DFI profiles obtained using the crystal structures we find that the LTE distrubtion broadens with increasing DFI and the mean LTE per bin appears to be positively correlated with increasing DFI. (*A*) shows violin plots between the LTE of the protein and the binned percentile DFI values of the combined crystal structures of thioredoxin and *β*-lactamase ancestors and in (*B*) we see the same for a larger dataset of 100 proteins listed in S1 Data.

Given that LTE follows a similar trend, we investigate further if LTE captures local flexibility in a comparable manner. It is important to note that while DFI measures the resistance of an amino acid position to perturbations at other sites (i.e., how much a position is influenced by Brownian kicks applied elsewhere in the protein), LTE is a purely local topological/geometrical metric through application on (static) protein structures. LTE quantifies the rarity of a specific local topological conformation formed by four consecutive residues in the protein chain relative to the PDB folded state ensemble. Despite this fundamental difference between the two metrics, our comparison of the LTE and DFI values of the combined crystal structures of thioredoxin and *β*-lactamase ancestors (Fig 3A) shows that positions with higher flexibility tend to have higher LTE. To test whether this correlation holds more generally, we examined a larger set of crystal structures (Fig 3B). To quantify the extent of the correlation we used the Spearman’s rank coefficent betwen the DFI bins and the variance of the LTE for that bin. We found the value to be 1 for 5 bins and even on increasing the bins to 20 the correlation was 0.88 with a p-value of $3\times 10^{-7}$. We observed that regions with higher LTE also tend to be more flexible, as reflected by a sharper peak at high LTE values and an extended high-LTE tail in the violin plot with increasing DFI. TEM-1 and GNCA also show a similar positive correlation between LTE and DFI (S3 Fig). One possible explanation for this correlation is that more flexible regions of the protein are better able to access the rarer writhe conformations associated with high LTE. These findings suggest that LTE—though derived solely from static structural data— is closely linked to the protein’s dynamic landscape and offers meaningful insights into its flexibility and motion [13,14,56].

### The evolution of the global topological landscape of proteins

Building on our observation that LTE, despite being a purely local structural metric, provides meaningful insights into protein dynamics, we now extend our analysis to global topological descriptors in the conformational ensemble. Specifically, we examine global writhe—a measure of structural entanglement computed from the full 3D conformation—using ensembles of structures sampled from molecular dynamics simulations of both ancestral and extant enzymes. The *β*-lactamase simulations used in our analysis were obtained from previously published work [53]. This approach allows us to assess whether global topological features, like the writhe of the entire protein, reflect evolutionary changes in protein flexibility and function.

**Fig 4:**
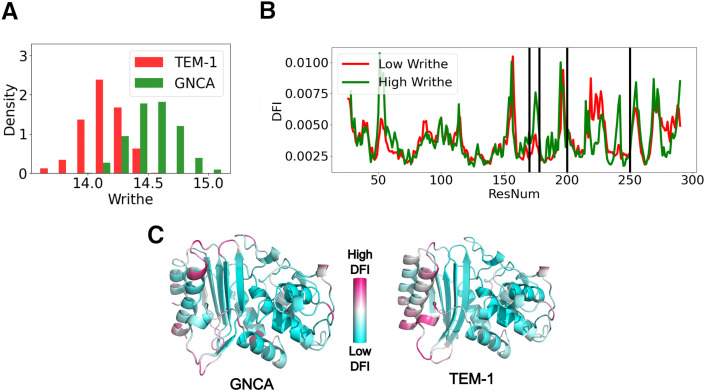
Writhe and the dynamical behavior of TEM-1. On calculating the global writhe of TEM-1 and the Gram-Negative Common Ancestor (GNCA) of *β*-lactamase from simulation data, we find that the writhe of the ancestral variant GNCA is significantly higher on average than that of TEM-1 and has higher variance as shown in **(*A*)**. This indicates that the extant protein adopts a more narrow conformational landscape and reflects a possible global tightening of a hinge movement. In (*B*) we see that the DFI profiles obtained from the low and the high writhe structures reveal key differences between residues 200-250 and also between 170-179 which corresponds to the functionally critical Ω loop of *β*-lactamase. In (*C*) we see the DFI profile colored on the structure.

To investigate the role of global topological metrics in protein evolution, we analyzed the global writhe distributions of the ancestral (GNCA) and extant (TEM-1) variants of *β*-lactamase. We note that since the two proteins have the same length, their writhe distributions are directly comparable. Using conformations sampled from molecular dynamics simulations, we computed the global writhe for both variants. The results, shown in Fig 4, reveal a statistically significant shift in the writhe distribution (P-value = $4\times 10^{-202}$ on performing a KL Divergence test to show statistically that the two distributions differ.), Fig 4A), with mean values of 14.53 for GNCA and 14.14 for TEM-1. Additionally, the variance of the distribution has slightly decreased (*σ* = 0.20 for GNCA and *σ* = 0.16 for TEM-1), indicating that the conformational landscape has become more restricted. This reduction in writhe and landscape variability aligns with our vibrational density of states analysis [72], which suggests that, as *β*-lactamases evolved from a generalist enzyme to a specialist in penicillin degradation, the native ensemble of TEM-1 shifted toward a more defined conformational states, shaping its structural dynamics for efficient substrate processing. The decrease in the average Writhe value indicates that the *β*-lactamase has evolved to a more compact conformation. The compactness of the structure, reflected by the higher Writhe can be either due to the better formation of longer helical structures or to the global tightening of a hinge movement. To explore the extremes of the global writhe distributions for TEM-1, we computed the DFI profiles for conformations belonging to the highest writhe bins of TEM-1 and the lowest writhe bins of GNCA. These profiles represent the extreme cases within these two distributions (Fig 4B). While the overall DFI profiles are similar, the most significant differences are observed in the regions between residues C*α* 200–250 and around residues 170–179, which correspond to the functionally critical Ω loop of the *β*-lactamase. Previous studies using both NMR [73] and MD simulations [74] have highlighted the crucial role of the dynamics of the Ω loop in the efficient degradation of various antibiotics.

Similarly, in the *E. coli* branch of thioredoxin, we observe a distinct distribution of global writhe between the LBCA and the extant *E. coli* (P-value = $1\times 10^{-202}$ obtained on performing a similar KL-divergence test as the one reported above), see panel A of S4 Fig. In particular, the variance of writhe is higher in the extant enzyme (*σ* = 0.16 for LBCA, *σ* = 0.25 for *E. coli*), suggesting an increase in conformational space for the modern homolog. Additionally, the average writhe of the extant thioredoxin (6.52) is lower than that of the ancestral form (6.88). These findings are consistent with evolutionary analyses of TEM-1, which suggest a shift in conformational ensembles during evolution. Moreover, when we compare the DFI profiles obtained from the conformational ensemble of the LBCA with higher writhe bins to the ensemble of *E. coli* with lower writhe bins (where the two distributions do not overlap), we find notable differences in the DFI profiles, particularly in the α4 region, as expected. Interestingly, the largest differences are observed in the regions preceding α4 and α3.

Previous studies have shown that comparing the differences in DFI scores between ancestral and extant thioredoxins in the human and *E. coli* branches provides insight into their adaptation to lower temperatures. In bacterial thioredoxins, the increased flexibility in the *α*3 region (resulting from hinge loss), which primarily contributes to stability, is compensated by a decrease in flexibility in *α*4, a region critical for folding. This trade-off may explain why modern thioredoxins exhibit reduced stability while preserving their canonical 3D fold [35]. This process, known as hinge shift, involves the relocation of a hinge (a position with low flexibility) from one region of the protein to another [1,35].

The increased flexibility of *α*3, as observed in [35], corresponds to greater conformational freedom relative to the rest of the protein within its conformational landscape. To examine how this increased flexibility influences the protein’s topological landscape, we analyze the intercrossing number (ICN) of *α*3 with the rest of the protein in both ancestral and extant variants. The ICN is employed to analyze the conformational complexity of two components with respect to each other (in this case the helix with the rest of the protein), as opposed to single conformational complexity that the writhe captures. We note that we employ the ICN instead of the linking number because the ICN captures subtle geometrical information that can be lost in the linking number (due to sign cancellations), which is more topological in nature.

**Fig 5:**
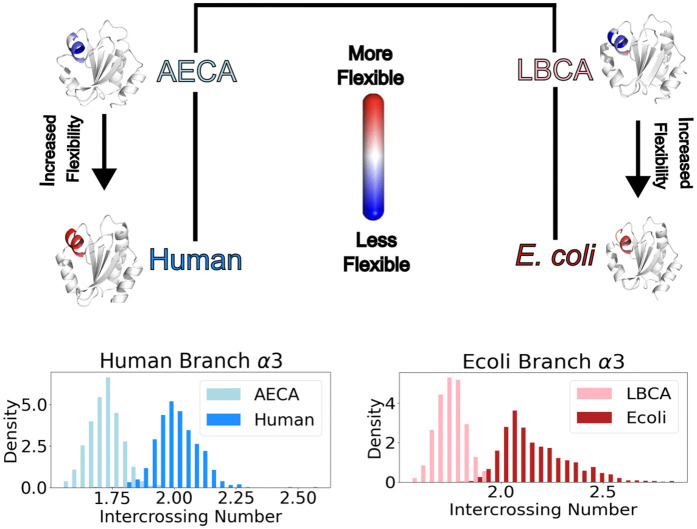
Flexibility correlates with the intercrossing number distribution. The *α*3 helix is highlighted, and it is observed to become more flexible in extant species. The distribution of the intercrossing number is also shown, with a larger variance observed in more flexible extant species. This increased variance is accompanied by a shift in the mean, where higher flexibility is correlated with a higher mean intercrossing number.

The results, shown in Fig 5, indicate that the ICN distribution in the extant protein is shifted toward higher mean values 2.16 for *E. coli* compared to 1.76 for LBCA and 2.01 for the extant human as compared to 1.73 for the ancestral variant. This is also accompanied by an increase in variance from $4.5\times 10^{-3}$ for AECA to $7.3\times 10^{-3}$ for the human variant. We see a similar increase in the variance for the *E. coli* branch with the variance changing from $4.9\times 10^{-3}$ for LBCA to $2.8\times 10^{-2}$ for the extant *E. coli* variant. The shift in ICN suggests that *α*3 has greater topological accessibility, providing insight into its enhanced flexibility in modern thioredoxin. This is further supported by the increased variance, which reflects the ability of *α*3 to explore a broader range of relative positions. Additionally, *α*3 appears to adopt conformations that interact more closely with the rest of the protein, as evidenced by the increase in mean ICN. This observation suggests that *α*3 may form a tighter turn or position itself within a structural “pocket” of the protein. Thus, this topological feature solely analyzed using molecular dynamics simulations based on crystal structures of ancestral and modern thioredoxin provides mechanistic insight about dynamics divergence of thioredoxin during their functional evolution.

For α4 we find that the variance of ICN decreases with evolution (see S5 Fig). This is in agreement with α4 having decreased flexibility, and thus being constraint to a narrower topological landscape.

## Discussion

It has been established via both experiments and simulations that structure-encoded dynamics governs function, yet, the understanding of the exact mechanisms of this connection remains elusive. An exceptional context within which the relation between structure and dynamics can be explored is that of protein evolution. Ancestrally reconstructed proteins have shown that the evolution of a new function and/or adaptation to a new environment is always accomplished while preserving the 3D structure.

In this work, we employ mathematical topology and geometry to rigorously characterize functional evolution within a protein family through extensive sequence diversification while maintaining essentially invariant three-dimensional structures by including ancestral and extant homologs. This design allows us to isolate whether topological metrics computed solely from structure, using only backbone C-*α* positions, can capture functional divergence that sequence-based analyses detect but traditional structure-based metrics cannot. Our analysis focuses on two well-studied systems: thioredoxin and *β*–lactamase. Specifically, we examine global structural properties using the Gauss linking integral. To capture local structural features, we employ the local topological free energy (LTE), which quantifies the rarity of a given local writhe value within the protein data bank (PDB) [75,76] ensemble of folded proteins.

Our results showed that the LTE of the static native state alone can distinguish the ancestral and extant structures and moreover, reconstruct the evolutionary tree. By comparing the LTE of both thioredoxin and *β*-lactamase proteins with their DFI profile, we find that LTE correlates with the DFI profile of a protein. This is the first time that LTE (a measure of native structure geometrical complexity) is associated with its dynamical response under perturbation.

By employing molecular dynamics simulations for both the ancestral and extant proteins (of both thioredoxin and *β*-lactamase), we obtain the topological landscape in writhe. Our results show that the extant and ancestral topological landscapes in writhe are different. For all systems, we observe that the DFI profiles for the extremes of the writhe distributions are different at sites important for the evolution of function. In order to better understand which aspects of the conformation contribute to the DFI profile differences, we focus on the α3 and α4 helices in thioredoxin, whose DFI profile indicates a transition to increased (resp. decreased) flexibility. By making use of the intercrossing number, which captures the relative topological/geometrical landscape of the helix with respect to the rest of the protein, we observe a shift in all systems (including the human branch), which indicates a larger (resp. smaller) ensemble of geometrical conformations with increasing (resp. decreasing) flexibility.

These results establish a direct link between the mathematical geometrical and topological complexity of proteins, seen as open curves in 3-space, and their dynamical properties. By uncovering this relationship, we provide a quantitative framework for analyzing protein structure that offers deeper insights into the interplay between structure, dynamics, and function.

## Supporting information

S1 Fig Crossings in diagrams. The projection of an oriented curve gives rise to crossings of two types: a positive crossing (Left) and a negative crossing (Right).
(PDF)

S2 Fig LTE analysis of TEM-1. Panel **A** shows the LTE profile colored on the structures of TEM-1 and GNCA. In **B** we see the LTE profiles cluster together based on how they evolve. The clustering was performed using the Ward’s linkage method taking the top three eigenvalues as they accounted for over 90% of the variance.
(PDF)

S3 Fig β-lactamase LTE vs DFI Profiles. A shows the correlation of TEM-1 with DFI and B shows the same for GNCA. We find a similar trend in the LTE vs percentile DFI for both TEM-1 and GNCA, i.e., higher LTE implies more flexibility.
(PDF)

S4 Fig Low vs High Writhe DFI profiles for the *E.Coli.* **A** shows the writhe distribution of extant *E. coli* and LBCA. We observe the same trend seen in TEM-1, namely that extant species exhibit lower writhe on average compared to their ancestors. In **B** we perform an analysis analogous to that in the main text and find that the flexibility of the *α*4 helix obtained from the high-writhe LBCA structures is greater than that obtained from the low-writhe *E. coli* structures. The simulation data used for this analysis is available at Zenodo (doi: https://doi.org/10.5281/zenodo.17716722).
(PDF)

S5 Fig Flexibility vs ICN for the *α*4 helix. In the main text we had seen that the variance of the intercrossing number of the *α*3 helix was correlated with its flexibility. We observe a similar correlation between the variance of the ICN distribution with that of the flexibility of the *α*4 helix.
(PDF)

S6 FigExamples of global and local Writhe.(A) Global Writhe (*Wr*) of PDB: 1A2P (resp. (B) 1A12, (C) 1A4I). In these examples, *Wr* increases in absolute value as the length and complexity of the protein increases. (D) Local Writhe values of PDB: 1A2P amino acids 31–34, (E) 1A12 amino acids 110–113, and (F) 1A4I amino acids 185–183. The local Writhe of an amino acid is the Writhe of a polygonal curve of three edges: in practice the Gauss linking integral between the first and third edge (since consecutive edges have zero linking number). This quantity equals the geometric probability that the two straight segments cross in any projection direction (divided by 2). Local writhe satisfies −1≤Wr≤1. It measures local orientation and compactness: a tight right-handed (resp. left-handed) turn yields values close to *1* (resp. −1), while straighter segments have values near 0.(PDF)

S7 Fig Examples of local conformations in the protein sample and their corresponding local Writhe values. A, B: Similar dihedral angles, different Writhe. C, D: Similar Writhe, different dihedral angles. (axes units are in Å).
(PDF)

S8 Fig Examples of local conformations in the protein sample with low vs high LTE. (A) low LTE, (B) high LTE > 2.6 (axes units are in Å).
(PDF)

S9 Fig Examples of different ICN. The intercrossing number (ICN) captures the complexity of the conformation of two chains relative to each other. In the manuscript, we employ the ICN to characterize the complexity of a conformation of a part of a protein relative to the rest of the protein. Here we show examples of the ICN between a region of the protein (green) and the remainder of the chain (red). The conformation in panel A has a higher ICN value compared to panel B, reflecting that the green region in panel A is more surrounded by and wrapped by the rest of the protein for a sample protein (PDB ID: 1V9E).
(PDF)

S1 Data Enzyme dataset. List of 100 PDB files used for validating the correlation between LTE and DFI.
(CSV)
