# Supplementary material for: Topological metrics as evolutionary and dynamical descriptors of conformational landscapes within protein families
Source: PLoS Comput Biol. 2026 Mar 4;22(3):e1013985. doi: 10.1371/journal.pcbi.1013985 (PMC12995304; doi:10.1371/journal.pcbi.1013985)
Supplement: S1 Fig — The projection of an oriented curve gives rise to crossings of two types: a positive crossing (Left) and a negative crossing (Right). (PDF) [file pcbi.1013985.s001.pdf]

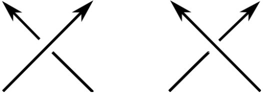

S1 Fig. Crossings in diagrams. The projection of an oriented curve gives rise to crossings of two types: a positive crossing (Left) and a negative crossing (Right).
