# Supplementary material for: Topological metrics as evolutionary and dynamical descriptors of conformational landscapes within protein families
Source: PLoS Comput Biol. 2026 Mar 4;22(3):e1013985. doi: 10.1371/journal.pcbi.1013985 (PMC12995304; doi:10.1371/journal.pcbi.1013985)
Supplement: S2 Fig — Panel A shows the LTE profile colored on the structures of TEM-1 and GNCA. In B we see the LTE profiles cluster together based on how they evolve. The clustering was performed using the Ward’s linkage method taking the top three eigenvalues as they accounted for over 90% of the variance. (PDF) [file pcbi.1013985.s002.pdf]

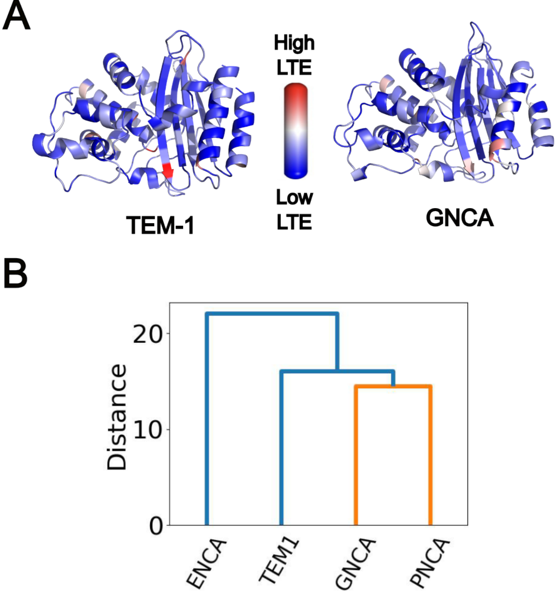

S2 Fig. LTE analysis of TEM-1.

Panel **A** shows the LTE profile colored on the structures of TEM-1 and GNCA. In **B** we see the LTE profiles cluster together based on how they evolve.
