# Supplementary material for: Topological metrics as evolutionary and dynamical descriptors of conformational landscapes within protein families
Source: PLoS Comput Biol. 2026 Mar 4;22(3):e1013985. doi: 10.1371/journal.pcbi.1013985 (PMC12995304; doi:10.1371/journal.pcbi.1013985)
Supplement: S3 Fig — A shows the correlation of TEM-1 with DFI and B shows the same for GNCA. We find a similar trend in the LTE vs percentile DFI for both TEM-1 and GNCA, i.e., higher LTE implies more flexibility. (PDF) [file pcbi.1013985.s003.pdf]

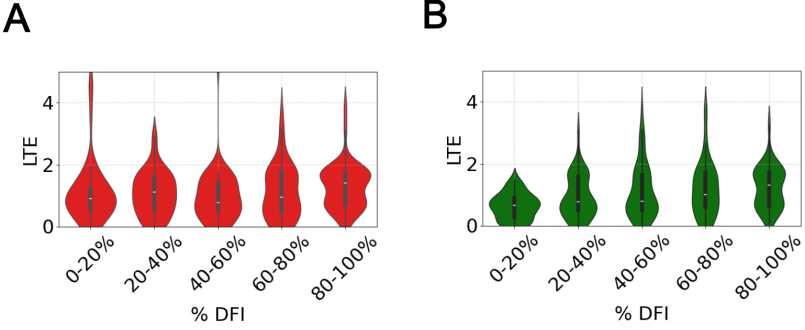

S3 Fig. β-lactamase LTE vs DFI profiles.

A shows the correlation of TEM-1 with DFI and **B** shows the same for GNCA. We find a similar trend in the LTE vs percentile DFI for both TEM-1 and GNCA, i.e., higher LTE implies more flexibility.
