# Supplementary material for: Topological metrics as evolutionary and dynamical descriptors of conformational landscapes within protein families
Source: PLoS Comput Biol. 2026 Mar 4;22(3):e1013985. doi: 10.1371/journal.pcbi.1013985 (PMC12995304; doi:10.1371/journal.pcbi.1013985)
Supplement: S4 Fig — A shows the writhe distribution of extant E. coli and LBCA. We observe the same trend seen in TEM-1, namely that extant species exhibit lower writhe on average compared to their ancestors. In B we perform an analysis analogous to that in the main text and find that the flexibility of the α4 helix obtained from the high-writhe LBCA structures is greater than that obtained from the low-writhe E. coli structures. The simulation data used for this analysis is available at Zenodo (doi: https://doi.org/10.5281/zenodo.17716722). (PDF) [file pcbi.1013985.s004.pdf]

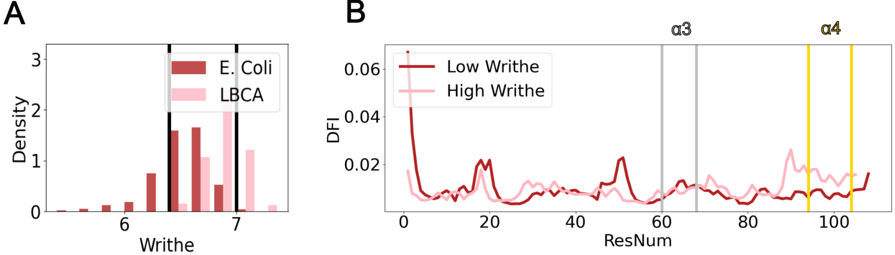

S4 Fig. Low vs High Writhe DFI profiles for the *E. coli* branch of Thioredoxin.

A shows the writhe distribution of extant *E. coli* and LBCA. We observe the same trend seen in TEM-1, namely that extant species exhibit lower writhe on average compared to their ancestors. In **B** we perform an analysis analogous to that in the main text and find that the flexibility of the $\alpha4$ helix obtained from the high-writhe LBCA structures is greater than that obtained from the low-writhe *E. coli* structures. The simulation data used for this analysis is available at Zenodo (doi: https://doi.org/10.5281/zenodo.17716722).
