# Supplementary material for: Topological metrics as evolutionary and dynamical descriptors of conformational landscapes within protein families
Source: PLoS Comput Biol. 2026 Mar 4;22(3):e1013985. doi: 10.1371/journal.pcbi.1013985 (PMC12995304; doi:10.1371/journal.pcbi.1013985)
Supplement: S5 Fig — In the main text we had seen that the variance of the intercrossing number of the α3 helix was correlated with its flexibility. We observe a similar correlation between the variance of the ICN distribution with that of the flexibility of the α4 helix. (PDF) [file pcbi.1013985.s005.pdf]

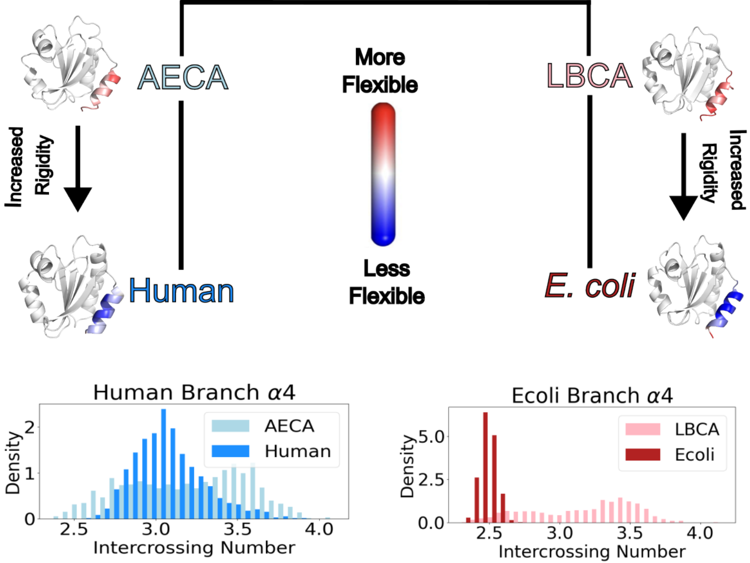

S5 Fig. Flexibility vs ICN for the $\alpha4$ helix.

In the main text we observed that the variance of the intercrossing number (ICN) of the $\alpha3$ helix was correlated with its flexibility. Here we observe a similar correlation between the variance of the ICN distribution and the flexibility of the $\alpha4$ helix.
