# Supplementary material for: Topological metrics as evolutionary and dynamical descriptors of conformational landscapes within protein families
Source: PLoS Comput Biol. 2026 Mar 4;22(3):e1013985. doi: 10.1371/journal.pcbi.1013985 (PMC12995304; doi:10.1371/journal.pcbi.1013985)
Supplement: S6 Fig — (A) Global Writhe (Wr) of PDB: 1A2P (resp. (B) 1A12, (C) 1A4I). In these examples, Wr increases in absolute value as the length and complexity of the protein increases. (D) Local Writhe values of PDB: 1A2P amino acids 31–34, (E) 1A12 amino acids 110–113, and (F) 1A4I amino acids 185–183. The local Writhe of an amino acid is the Writhe of a polygonal curve of three edges: in practice the Gauss linking integral between the first and third edge (since consecutive edges have zero linking number). This quantity equals the geometric probability that the two straight segments cross in any projection direction (divided by 2). Local writhe satisfies −1≤Wr≤1. It measures local orientation and compactness: a tight right-handed (resp. left-handed) turn yields values close to 1 (resp. −1), while straighter segments have values near 0. (PDF) [file pcbi.1013985.s006.pdf]

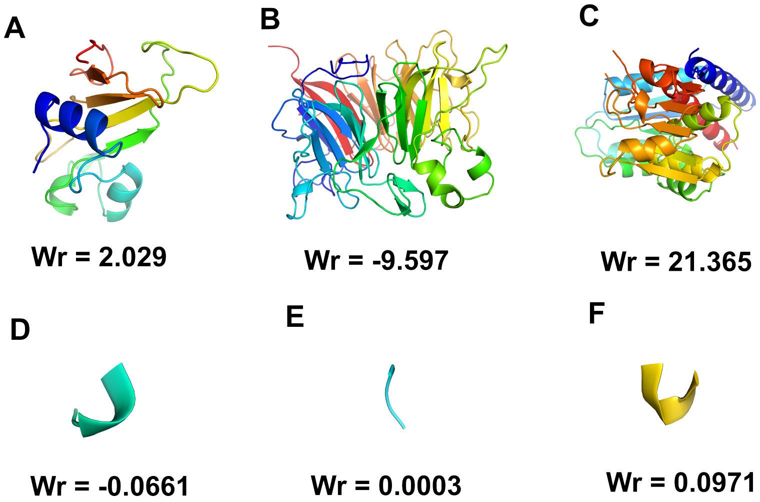

S6 Fig. Examples of global and local writhe.

(A) Global Writhe (Wr) of PDB: 1A2P (resp. (B) 1A12, (C) 1A4I). In these examples, Wr increases in absolute value as the length and complexity of the protein increases. (D) Local Writhe values of PDB: 1A2P amino acids 31-34, (E) 1A12 amino acids 110-113, and (F) 1A4I amino acids 185-183. The local Writhe of an amino acid is the Writhe of a polygonal curve of three edges: in practice the Gauss linking integral between the first and third edge (since consecutive edges have zero linking number). This quantity equals the geometric probability that the two straight segments cross in any projection direction (divided by 2). Local writhe satisfies $-1 \leq Wr \leq 1$. It measures local orientation and compactness: a tight right-handed (resp. left-handed) turn yields values close to 1 (resp. -1), while straighter segments have values near 0.
