# Supplementary material for: Topological metrics as evolutionary and dynamical descriptors of conformational landscapes within protein families
Source: PLoS Comput Biol. 2026 Mar 4;22(3):e1013985. doi: 10.1371/journal.pcbi.1013985 (PMC12995304; doi:10.1371/journal.pcbi.1013985)
Supplement: S7 Fig — A, B: Similar dihedral angles, different Writhe. C, D: Similar Writhe, different dihedral angles. (axes units are in Å). (PDF) [file pcbi.1013985.s007.pdf]

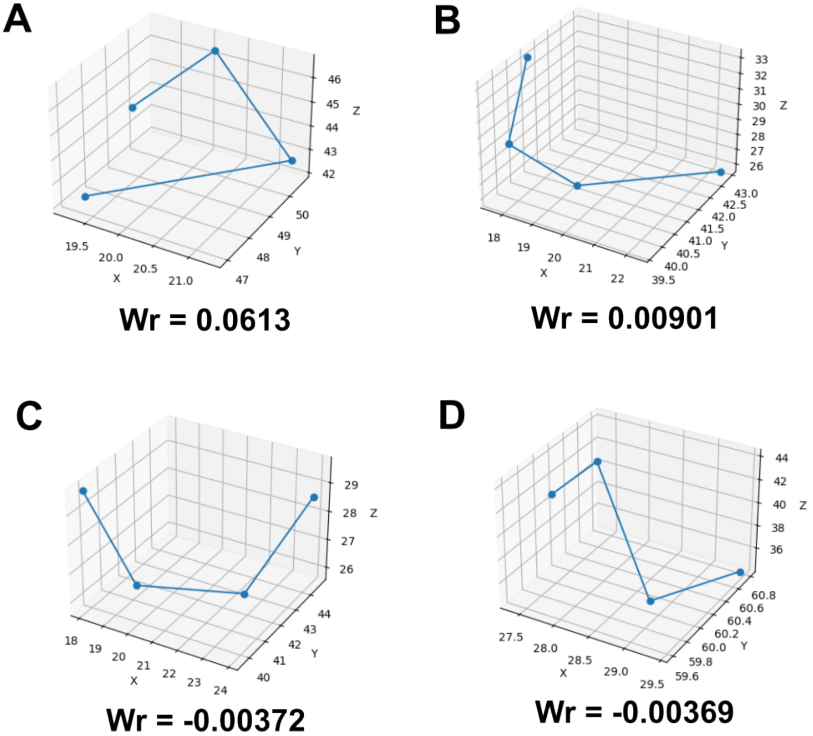

S7 Fig. Examples of local conformations and their local Writhe values.

Examples of local conformations in the protein sample and their corresponding local Writhe values (axes units in Å). **A, B**: Similar dihedral angles, different Writhe. **C, D**: Similar Writhe, different dihedral angles.
