# Supplementary material for: Topological metrics as evolutionary and dynamical descriptors of conformational landscapes within protein families
Source: PLoS Comput Biol. 2026 Mar 4;22(3):e1013985. doi: 10.1371/journal.pcbi.1013985 (PMC12995304; doi:10.1371/journal.pcbi.1013985)
Supplement: S8 Fig — (A) low LTE, (B) high LTE > 2.6 (axes units are in Å). (PDF) [file pcbi.1013985.s008.pdf]

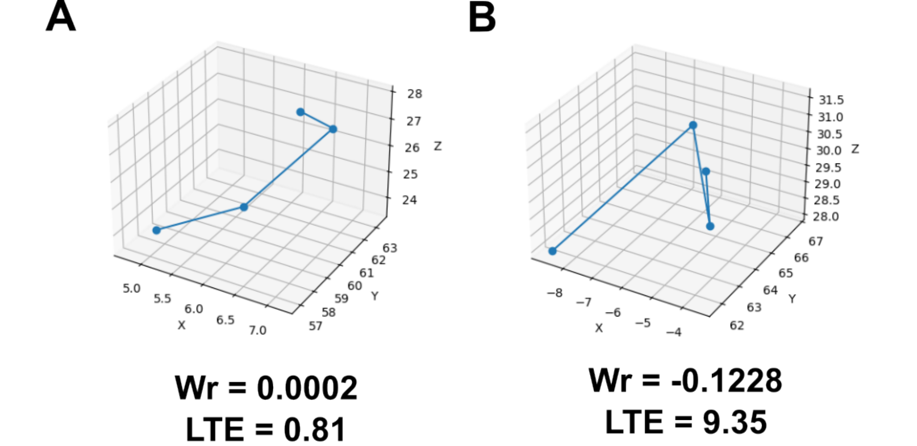

S8 Fig. Examples of local conformations with low vs high LTE.

Examples of local conformations in the protein sample with **(A)** low LTE and **(B)** high LTE > 2.6 (axes units in $\mathring{A}$).
