# Supplementary material for: Topological metrics as evolutionary and dynamical descriptors of conformational landscapes within protein families
Source: PLoS Comput Biol. 2026 Mar 4;22(3):e1013985. doi: 10.1371/journal.pcbi.1013985 (PMC12995304; doi:10.1371/journal.pcbi.1013985)
Supplement: S9 Fig — The intercrossing number (ICN) captures the complexity of the conformation of two chains relative to each other. In the manuscript, we employ the ICN to characterize the complexity of a conformation of a part of a protein relative to the rest of the protein. Here we show examples of the ICN between a region of the protein (green) and the remainder of the chain (red). The conformation in panel A has a higher ICN value compared to panel B, reflecting that the green region in panel A is more surrounded by and wrapped by the rest of the protein for a sample protein (PDB ID: 1V9E). (PDF) [file pcbi.1013985.s009.pdf]

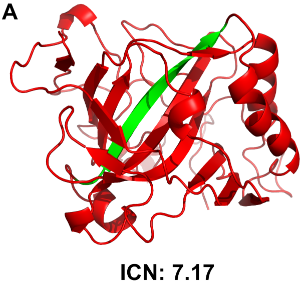

S9 Fig. Examples of different ICN.

The intercrossing number (ICN) captures the complexity of the conformation of two chains relative to each other. In the manuscript, we employ the ICN to characterize the complexity of a conformation of a part of a protein relative to the rest of the protein. Here we show examples of the ICN between a region of the protein (green) and the remainder of the chain (red). The conformation in panel **A** has a higher ICN value compared to panel **B**, reflecting that the green region in panel A is more surrounded by and wrapped by the rest of the protein for a sample protein (PDB ID : 1V9E).
